# 

**Published:** 1878-04

**Authors:** 


### CONTENTS:

Article I.—What Anæsthetics Shall We Use . . . . . 529

Article II.—Gingivitis—Its Different Forms—Treatment, &c. . . . . . 547

Article III—Plastic Filling, and the Basal Principles of the New Departure. . . . . 556

Article IV.—The So-called Riggs' Disease and its Treatment. . . . . 563

### EDITORIAL, ETC.

The Thirty-Eighth Annual Commencement of the Baltimore College of Dental Surgery . . . . . 567
Chemistry for Dental Students . . . . . 568
The Dental and Oral Science Magazine . . . . . 569

### OBITUARY.

Dr. George J. Barclay . . . . . 569

### MONTHLY SUMMARY.

Is the Human Eye Changing its Form, &c, under the Influence of Modern Education. 570

Relief for Foreign Bodies in the Ear . . . . . 571

Danger of Ether . . . . . 571

Injury from Disturbed Sleep . . . . . 572

A New Counter-Irritant . . . . . 572

Death from a Carious Tooth . . . . . 573

A Simple Telephone . . . . . 573

Calculus in the Sublingual Duct . . . . . 573

Results of Intermarriage . . . . . 574

American Dentists in Europe . . . . . 574

New Anæsthetic . . . . . 574

The Purity of Chloral . . . . . 575

Death from Chewing Tobacco . . . . . 575

Transplantation of Teeth . . . . . 575

Eucalyptus as a Local Anæsthetic . . . . . 575

Cyanide of Zinc in Facial Neuralgia . . . . . 576

Mixed Narcosis . . . . . 576

